# Human hepatocyte-derived extracellular vesicles attenuate the carbon tetrachloride-induced acute liver injury in mice

**DOI:** 10.1038/s41419-021-04204-7

**Published:** 2021-10-27

**Authors:** Masatoshi Kakizaki, Yuichiro Yamamoto, Shunya Nakayama, Kazuaki Kameda, Etsuko Nagashima, Masatoshi Ito, Takashi Suyama, Yumi Matsuzaki, Tetsuhiro Chiba, Hideaki Sumiyoshi, Yutaka Inagaki, Ai Kotani

**Affiliations:** 1 Department of Innovative Medical Science, Tokai University School of Medicine, Kanagawa, 259-1193 Japan; 2 Division of Hematological Malignancy, Institute of Medical Sciences, Tokai University, Kanagawa, 259-1193 Japan; 3 Division of Hematology, Jichi Medical University Saitama Medical Center, Saitama, 330-8503 Japan; 4 Support Center for Medical Research and Education, Tokai University School of Medicine, Kanagawa, 259-1193 Japan; 5 Department of Life Science, Shimane University Faculty of Medicine, Izumo, Shimane Japan; 6 Department of Gastroenterology, Chiba University, Graduate School of Medicine, Inohana, Chuo-ku, Chiba, 260-8670 Japan; 7 Center for Matrix Biology and Medicine, Graduate School of Medicine, Tokai University, Kanagawa, 259-1193 Japan; 8 Institute of Medical Sciences, Tokai University, Kanagawa, 259-1193 Japan

**Keywords:** Acute inflammation, Liver diseases

## Abstract

Acute liver injury (ALI) induced by chemicals or viruses can progress rapidly to acute liver failure (ALF), often resulting in death of patients without liver transplantation. Since liver transplantation is limited due to a paucity of donors, expensive surgical costs, and severe immune rejection, novel therapies are required to treat liver injury. Extracellular vesicles (EVs) are used for cellular communication, carrying RNAs, proteins, and lipids and delivering them intercellularly after being endocytosed by target cells. Recently, it was reported that EVs secreted from human hepatocytes have an ability to modulate the immune responses; however, these roles of EVs secreted from human hepatocytes were studied only with in vitro experiments. In the present study, we evidenced that EVs secreted from human hepatocytes attenuated the CCL_4_-induced ALI by inhibiting the recruitment of monocytes through downregulation of chemokine receptor in the bone marrow and recruitment of neutrophils through the reduction of C-X-C motif chemokine ligand 1 (CXCL1) and CXCL2 expression levels in the liver.

## Introduction

The liver is a highly vascularized large organ responsible for the metabolism of carbohydrates, proteins, and lipids, as well as removal of drugs and toxins from the blood. Moreover, it acts as a key frontline immune tissue. While the liver’s default immune status is anti-inflammatory or immunotolerant due to continuous exposure to large amounts of circulating antigens derived from food and gut microbiota via portal vein, it needs a rapid and robust immune response against exogenous antigens and endotoxins from gut microbiota [1]. Due to the complex immune status and the environment surrounding the liver, it is often exposed to various threats. Acute liver injury (ALI) induced by chemicals or viruses can progress rapidly to acute liver failure (ALF), often resulting in the death of patients without liver transplantation [2]. Since liver transplantation is limited due to a paucity of donors, expensive surgical costs, and severe immune rejection, novel therapies are required to treat ALI. ALF is a process of hepatocyte injury that is dominated by inflammatory reactions. Various hepatotoxic factors, such as concanavalin A (ConA) [3–5], acetaminophen (APAP) [3, 6], and lipopolysaccharide (LPS) [7] induce immune dysfunction in liver and then lead to ALF. Imbalance of the immune response plays a crucial role in the pathological process of ALF [8].

Recently, accumulating evidence have revealed that extracellular vesicles (EVs) secreted from mesenchymal stem cells (MSCs), with multilineage differentiation potential, self-renewal ability, and immunomodulatory functions or hepatocytes can effectively treat ALF by regulating the inflammatory responses [9–12].

Furthermore, EVs are secreted from various cell types to the extracellular space and they originate either from the plasma membrane or from the multivesicular bodies (MVBs) [13, 14]. EVs derived from MVBs are termed exosomes. The vesicles carry various biomacromolecules, such as proteins, mRNA, microRNA, and other noncoding RNAs [15, 16]. EVs not only trigger downstream signals but also transfer genetic material to the target cells, thereby exerting anti-inflammatory, antiapoptotic, and immunosuppressive effects, and thus, promote tissue repair and improve cytokine levels [17, 18]. EVs secreted from MSCs alleviate immune responses by reducing the expression levels of inflammatory cytokines such as interleukin (IL)-1β, IL-6, and tumor necrosis factor (TNF)-α, which improved liver function and reduced mortality in fulminant hepatic failure mice [9].

EVs secreted from normal hepatocytes of mice modulate toxin-associated gene expression leading to therapeutic outcomes including suppression of fibrogenesis, hepatocyte damage, and inflammation in mouse liver fibrosis [12]. Although EVs secreted from primary human hepatocytes also have an ability to modulate the immune responses in vitro [19], their roles are yet-to-be validated by in vivo experiments. Hence, it is necessary to investigate whether EVs secreted from human hepatocytes exert therapeutic effects using a mouse model.

In the present study, we demonstrated that the hepatoprotective effects of EVs secreted from human hepatocytes were markedly potent in the carbon tetrachloride (CCL_4_)-induced ALI mouse model. The EVs secreted from human hepatocytes attenuated the CCL_4_-induced ALI by inhibiting the recruitment of neutrophils to the liver via reduction of C-X-C motif chemokine ligand 1 (CXCL1) and CXCL2 in the liver and that of monocytes via reduction of chemokine receptor (CCR)-8 and CCR-9 in bone marrow (BM) cells possibly mediated by docosahexaenoic acid (DHA), thus producing potent anti-inflammatory and proresolutive lipid mediators, were enriched with EVs than with hepatocytes.

## Results

### The EVs secreted from human hepatocytes were protective

**Fig. 1:**
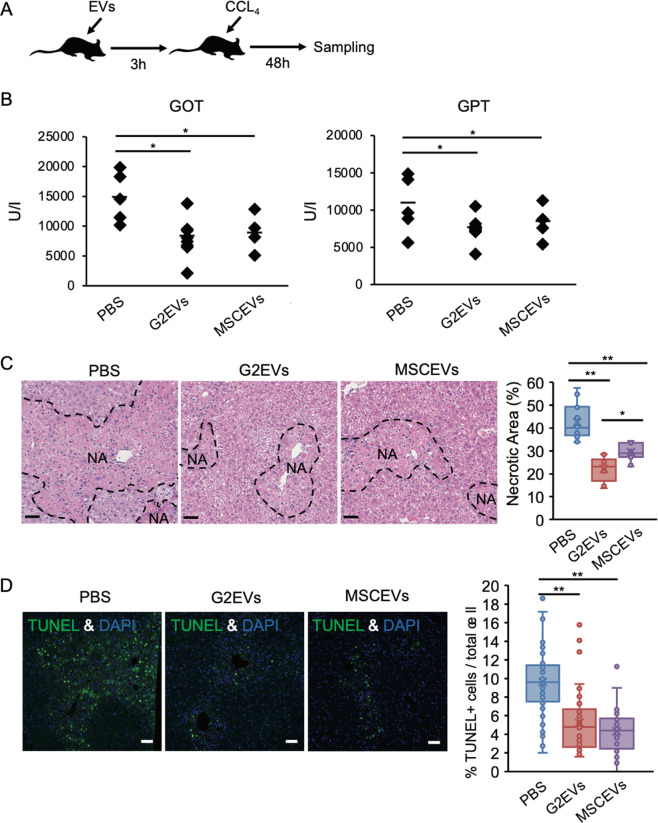
HepG2-EVs were more protective than the MSC-EVs for CCL_4_-induced acute liver injury (ALI). **A** Timeline for experiment of EV treatment for CCL_4_-induced ALI in mouse model. **B** The serum GOT and GPT were measured for the mice treated with CCL_4_ + PBS, CCL_4_ + HepG2-EVs, or CCL_4_ + MSC-EVs. **C** HE staining and **D** TUNEL staining of liver sections of the mice treated with CCL_4_ + PBS, CCL_4_ + HepG2-EVs, or CCL_4_ + MSC-EVs. **C** Areas of necrotic area (NA) were calculated in 5 fields from 3 mice. **D** TUNEL positive apoptotic cells (green) and DAPI positive nuclei (blue) are illustrated. TUNEL positive cells were enumerated in 10 fields from 3 mice. Ratio to the total cells was calculated. Statistical analysis was performed using one-way ANOVA and subsequent Tukey’s HSD method. *P < 0.05, **P < 0.01. Black and white bars indicate 50 μm.

Numerous studies revealed that the EVs secreted from MSC (MSC-EVs) induce potent immunomodulation and tissue regeneration [9], which are promising therapeutics. Moreover, it was reported that MSC-EVs ameliorate CCL_4_-induced ALI [9]. To test the hepatoprotective effects of the EVs collected from the culture supernatants of HepG2 cells (HepG2-EVs) in vivo, the EVs or MSC-EVs were injected to CCL_4_-induced liver injury mice. Timeline for the experiment of EV treatment for CCL_4_-induced ALI in a mouse model is illustrated in Fig. 1A. Both mice treated with MSC-EVs and those with HepG2-EVs demonstrated significant lower serum GOT and GPT in the serum and less TUNEL-positive cells in the liver than the mice treated with PBS (Fig. 1B, D). Importantly, the necrotic area in the liver with HepG2-EVs was significantly smaller than that with MSC-EVs (Fig. 1C).

**Fig. 2:**
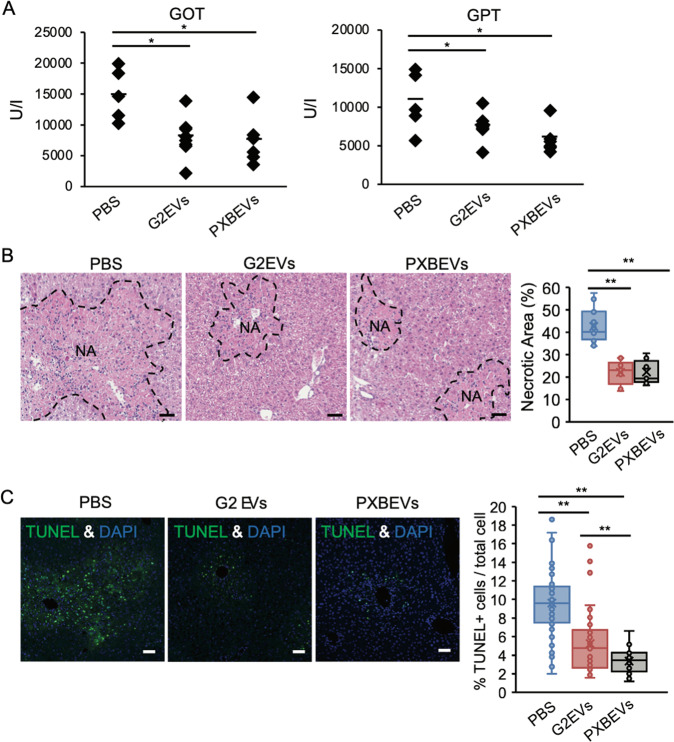
Hepatocyte-derived EVs were protective for the CCL_4_-induced acute liver injury. **A** The serum GOT and GPT were measured for the mice treated with CCL_4_ + PBS, CCL_4_ + HepG2-EVs, or CCL_4_ + PXB-EVs. **B** HE staining and **C** TUNEL staining of liver sections of the mice treated with CCL_4_ + PBS, CCL_4_ + HepG2-EVs, or CCL_4_ + PXB-EVs. **B** Areas of necrotic area (NA) were calculated in 5 fields from 3 mice. **C** TUNEL positive apoptotic cells (green) and DAPI positive nuclei (blue) are illustrated. TUNEL positive cells were enumerated in 10 fields from 3 mice. Ratio to total cells was calculated. Statistical analysis was performed using one-way ANOVA and subsequent Tukey’s HSD method. *P < 0.05, **P < 0.01. Black and white bars indicate 50 μm.

Furthermore, the effects observed in mice treated with HepG2-EVs were consistent with those in mice treated with EVs secreted from PXB cells (PXB-EVs). In addition, both mice treated with HepG2-EVs and PXB-EVs displayed significantly lower serum GOT and GPT than the control mice (Fig. 2A). The necrotic area and TUNEL-positive cells in the liver with HepG2-EVs or PXB-EVs treatment were significantly smaller than those in the control (Fig. 2B, C). These results suggest that EVs secreted from hepatocytes attenuate CCL_4_-induced ALI.

These EVs were characterized using transmission electron microscopy (TEM), tunable resistive pulse sensing (TRPS), and western blotting. They exhibited typical exosome-like round morphology, as observed by TEM (Fig. S1A). The TRPS analysis of these EVs by qNano nanoparticle analyzer indicated that the vesicles were ~100 nm in diameter (Fig. S1B). The presence of CD63 which is common EV/exosome marker was verified by western blotting (Fig. S1C).

Moreover, the therapeutic effects were also tested. Timeline of EV treatment for CCL_4_-induced ALI in a mouse model is illustrated in Supplementary Figure 2A. The mice treated with HepG2-EVs after addition of CCL_4_ demonstrated significant lower serum GOT and GPT in the serum than those treated with PBS (Fig. S2B), though the magnitude in these mice seemed less than in those treated with HepG2-EVs before addition of CCL_4_. These results indicate that the EVs from hepatocytes have therapeutic effects on CCL_4_-induced ALI.

### Liver resident macrophages are responsible for the attenuation of CCL_4_-induced ALI by the EVs secreted from human hepatocytes

**Fig. 3:**
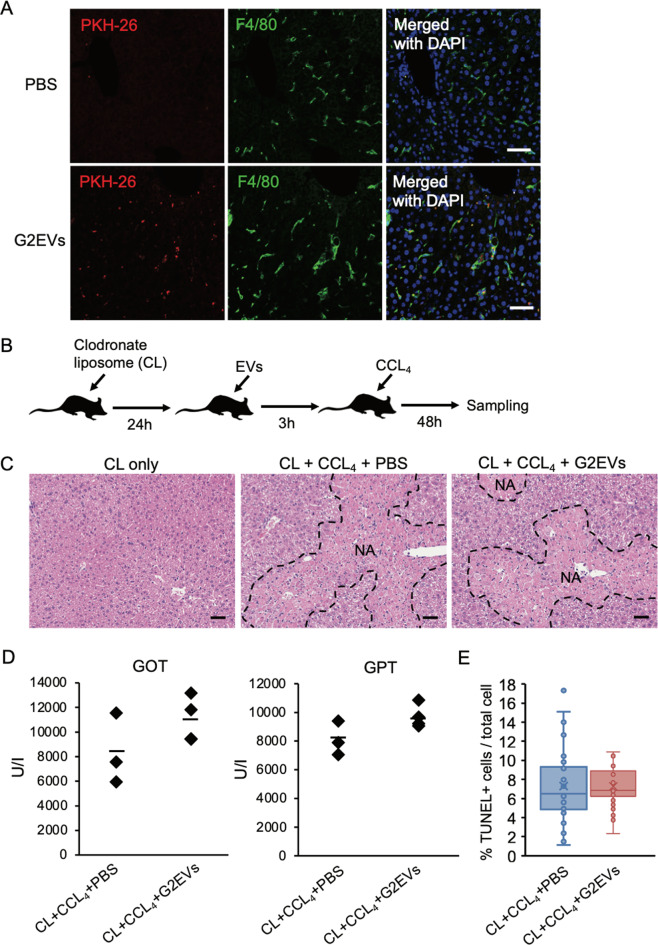
Liver resident macrophages are responsible for protective effects of hepatocyte-derived EVs. **A** F4/80 (green) and DAPI (blue) staining of liver sections of the mice treated with PBS or PKH-26 labeled HepG2-EVs (red). **B** Timeline for the experiment of CL and EV treatments for CCL_4_-induced acute liver injury in mouse model. **C** HE staining of liver sections of the mice treated with CL + CCL_4_ + PBS or CL + CCL_4_ + HepG2-EVs. **D** The Serum GOT and GTP were measured for the mice treated with CL + CCL_4_ + PBS or CL + CCL_4_ + HepG2-EVs. **E** TUNEL staining of liver sections of the mice treated with CL + CCL_4_ + PBS or CL + CCL_4_ + HepG2-EVs. TUNEL positive cells were enumerated in 10 fields from 3 mice. Ratio to total cells was calculated. Statistical analysis was performed using student’s *t* test. Black and white bars indicate 50 μm. CL clodronate liposome.

To investigate the target cells, which incorporate human hepatocyte-derived EVs in the liver, PKH26-labeled HepG2-EVs were injected into the tail vein of mice, and after 24 h, liver cryosections were prepared and examined via confocal laser microscopy. Thereafter, F4/80 positive cells, liver resident macrophages, mainly incorporated the EVs (Fig. 3A), suggesting that macrophages are involved in the attenuation of CCL_4_-induced ALI. The hypothesis was verified by depleting macrophages using clodronate liposomes (CL), which specifically eliminated the macrophages in CCL_4_-induced ALI (Fig. 3B). None of the necrotic area, serum GOT and GPT, or the ratio of TUNEL-positive cells differed between the mice treated with HepG2-EVs and the control under CL treatment (Fig. 3C–E). These results indicated that the EVs attenuate the CCL_4_-induced ALI through the macrophages incorporating EVs.

### Profile of the expression of proinflammatory cytokines and chemokines in the Kupffer cells

**Fig. 4:**
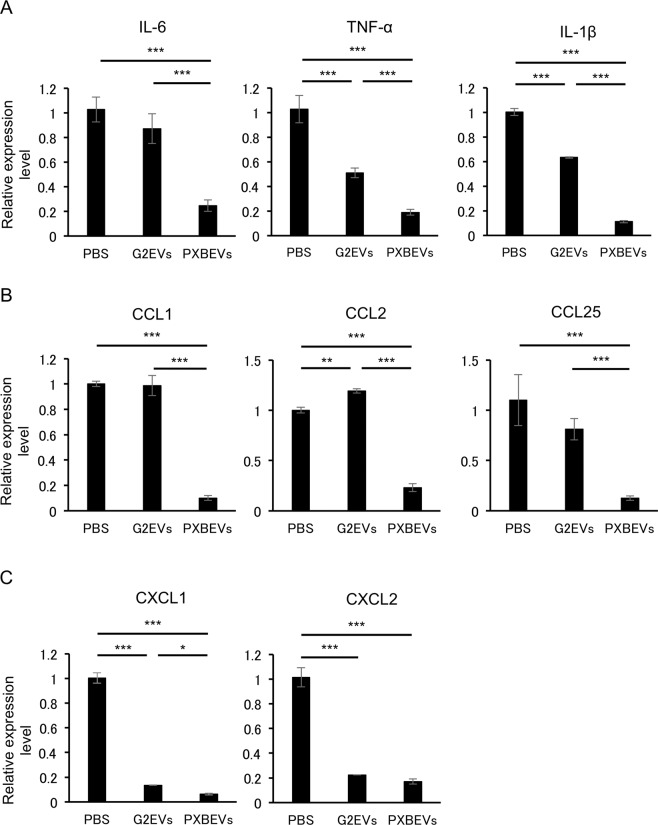
Hepatocyte-derived EVs affected the expression of proinflammatory cytokines and chemokines of KCs. **A**–**C** IL-1β, IL-6, TNF-α, CCL1, CCL2, CCL25, CXCL1, CXCL2, MMP-9, and MMP-12 mRNA expression in KCs of mice treated with CCL_4_ + PBS, CCL_4_ + HepG2-EVs, or CCL_4_ + PXB-EVs were measured by reverse-transcription quantitative polymerase chain reaction (RT-qPCR), and each sample was normalized relative to β-actin expression. Data represent the mean ± SD of triplicate experiments. Statistical analysis was performed using one-way ANOVA and subsequent Tukey’s HSD method. *P < 0.05.

In the CCL_4_-induced liver injury, Kupffer cells (KCs) are activated by detecting liver damage via damage-associated molecular patterns (DAMPs) released from hepatocytes. Thereafter, the activated KCs initiate inflammatory cascades [20]. Activated KCs secrete numerous chemokines and cytokines, resulting in further recruitment of leukocytes (e.g., monocytes and neutrophils) to the site of inflammation [20]. The KCs were purified from the liver in the CCL4 induced ALI mice with EV treatment (Supplemental Fig. 3A, B). The mRNA expression levels of proinflammatory cytokines, interleukin (IL)-1β and tumor necrosis factor (TNF)-α of the KCs in the mice treated with HepG2-EVs or PXB-EVs were significantly less than those with PBS. The expression of IL-6 tended to reduce in the mice treated with HepG2-EVs and significantly reduced in those with PXB-EVs (Fig. 4A). The mRNA expression levels of chemokines involved in monocyte migration, C-C motif chemokine ligand 1 (CCL1), CCL2, and CCL25 of the KCs in the mice treated with HepG2-EVs were almost the same as the those with PBS, whereas those with PXB-EVs were significantly less than those with HepG2-EVs and those with PBS (Fig. 4B). The mRNA expression levels of chemokines involved in neutrophil migration, C-X-C motif chemokine ligand 1 (CXCL1), and CXCL2 significantly reduced (Fig. 4C) in those with HepG2-EVs as well as those with PXB-EVs. These results indicate that proinflammatory cytokines are downregulated and the recruitment of neutrophil is suppressed by the EVs secreted from hepatocytes.

The effects of the EVs on the tissue regeneration were also investigated by analyzing the expression of matrix metalloproteinase (MMP) 9 and 12 in the liver. MMP-9 and MMP-12 were downregulated by both HepG2-EVs and PXB-EVs to certain extent (Fig. S4).

### Gr-1 positive neutrophils and F4/80 positive macrophage reduced in the liver of mice treated with EVs from hepatocytes

**Fig. 5:**
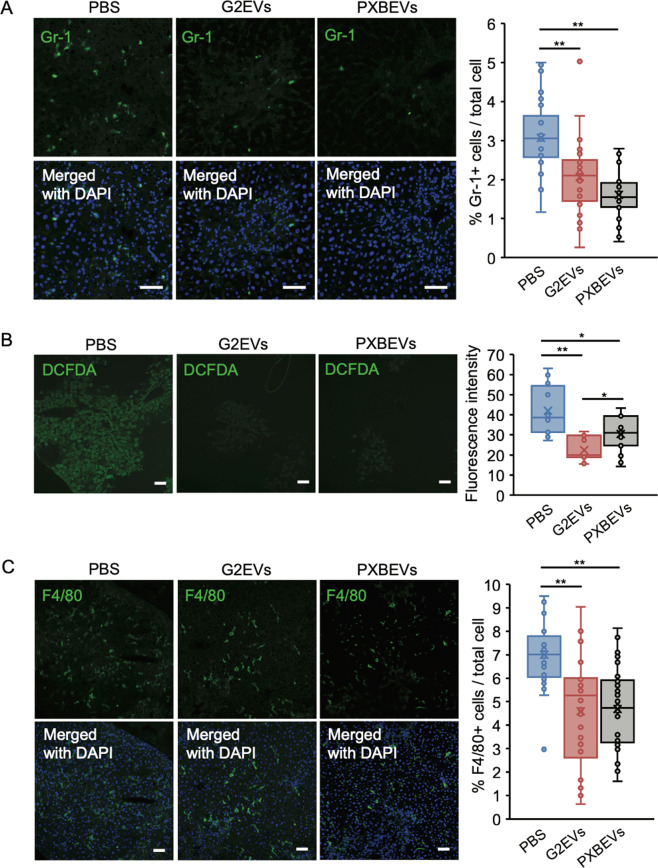
Hepatocyte-derived EV treatment reduced Gr-1 positive neutrophil and F4/80 positive cell infiltration into the liver. **A** Gr-1 (green) and DAPI (blue) staining of liver sections of the mice treated with CCL_4_ + PBS, CCL_4_ + HepG2-EVs, or CCL_4_ + PXB-EVs. Gr-1 positive cells were enumerated in 10 fields from 3 mice. Ratio to total cells was calculated. **B** ROS (green) staining using 2′,7′-dichlorofluorescenin diacetate (DCFDA) and DAPI (blue) staining of liver sections of the mice treated with CCL_4_ + PBS, CCL_4_ + HepG2-EVs, or CCL_4_ + PXB-EVs. Fluorescent intensity was measured in 10 fields from 3 mice using ImageJ. **C** F4/80 (green) and DAPI (blue) staining of liver sections of the mice treated with CCL_4_ + PBS, CCL_4_ + HepG2-EVs, or CCL_4_ + PXB-EVs. F4/80 positive cells were calculated in 10 fields from 3 mice. Ratio to total cells was calculated. Statistical analysis was performed using one-way ANOVA and subsequent Tukey’s HSD method. *P < 0.05, **P < 0.01. White bars indicate 50 μm.

The infiltrating neutrophils were examined in the liver of mice treated with HepG2-EVs, PXB-EVs, and PBS using immunofluorescence. The number of Gr-1 positive infiltrating neutrophils in the liver with HepG2-EVs or PXB-EVs treatment significantly reduced (Fig. 5A). Moreover, the amount of reactive oxygen species (ROS) which are mainly produced by the neutrophil markedly decreased in the liver of mice treated with HepG2-EVs and PXB-EVs compared to that with PBS (Fig. 5B).

The infiltrating monocytes were examined in the liver of mice treated with HepG2-EVs, PXB-EVs, and PBS using immunofluorescence. The number of F4/80 positive cells significantly reduced in the liver of mice treated with HepG2-EVs and PXB-EVs compared to that with PBS (Fig. 5C).

### Chemokine receptor of the bone marrow monocytes was significantly downregulated in the mice treated with the EVs from hepatocytes

Previously, we reported that the EVs derived from HepG2 cells replicating hepatitis B virus (HBV) are incorporated not only by KC in the liver but also by bone marrow monocytes (BMMC), which mainly migrated to the intestine to exhibit immunoregulatory functions [21]. We found that the EVs derived from HepG2 cells without replication of HBV are incorporated by BMMC although they are lesser than those from HepG2 replicating HBV.

**Fig. 6:**
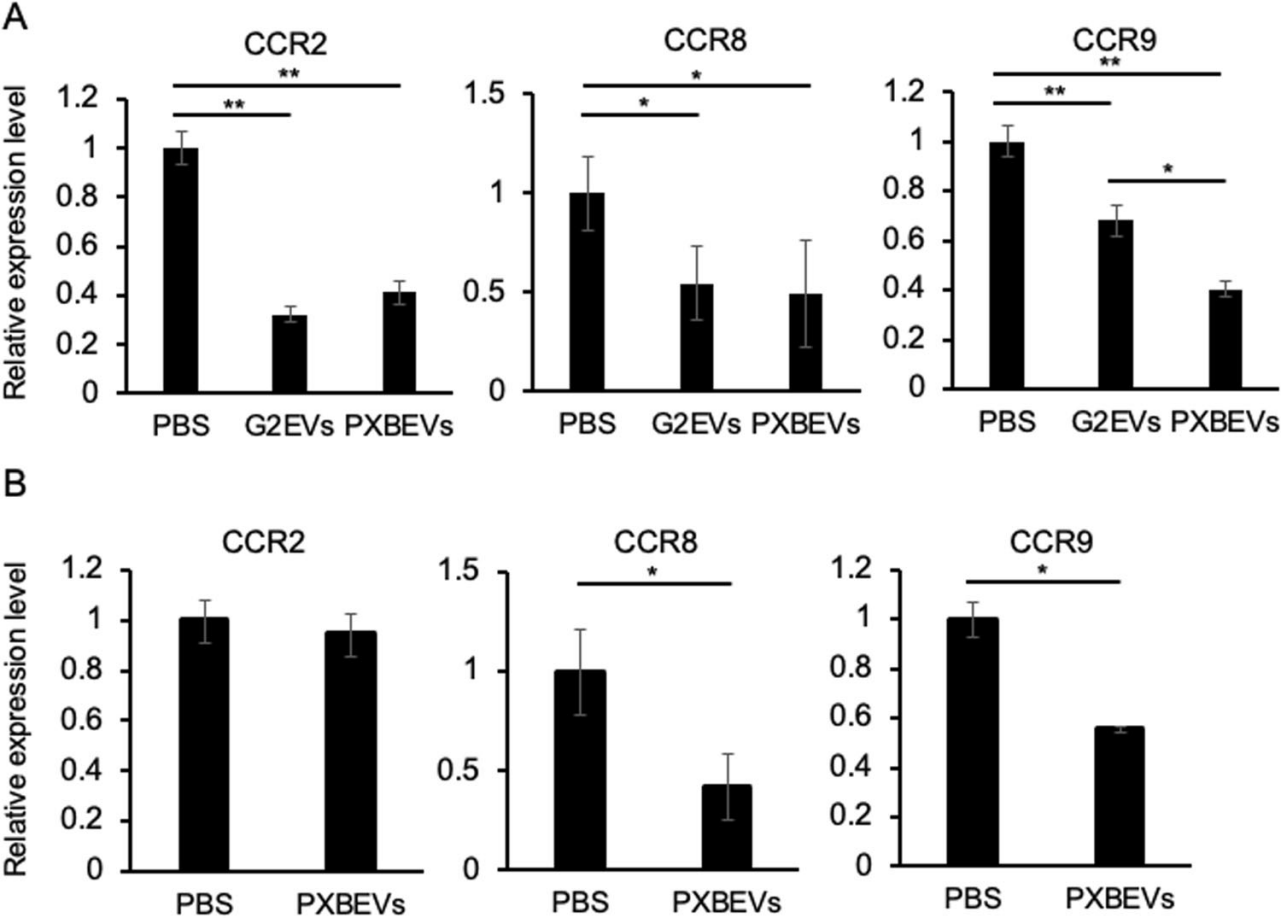
Hepatocyte-derived EV treatment reduced CCR2, CCR8, and CCR9 expression in BM cells. **A** CCR2, CCR8, and CCR9 mRNAs in BM cells 12 h after HepG2-EVs or PXB-EVs addition into culture supernatant were measured via real-time (RT-qPCR), and each sample was normalized relative to β-actin expression. Data represent the mean ± SD of triplicate experiments. **B** CCR2, CCR8, and CCR9 mRNA in BM cells collected from mice 48 h after injection of CCL_4_ + PBS or CCL_4_ + PXB-EVs were quantified using RT-qPCR. β-actin was used as an internal control. Data represent the mean ± SD of triplicate experiments. Statistical analysis was performed using one-way ANOVA and subsequent Tukey’s HSD method or student’s *t* test. *P < 0.05, **P < 0.01.

Thereafter, it was investigated whether the EVs secreted from hepatocytes change the expression levels of C–C chemokine receptor (CCR)2, CCR8, and CCR9, which are receptors of the CCL2, CCL1, and CCL25, respectively, in the BM cell. CCR2, CCR8, and CCR9 were significantly downregulated 12 h after EV treatment (Fig. 6A). Furthermore, in the BM cells collected from mice treated with PXB-EVs compared to those of the control, CCR8 and CCR9 expression levels significantly reduced (Fig. 6B). These results indicate that the accumulation of monocytes from BM might be inhibited due to reduction in the EV-induced CCR8 and CCR9 expression levels.

### Enrichment of DHA on the EVs was important for reducing CCR8 and CCR9 expression levels in BM cells

**Fig. 7:**
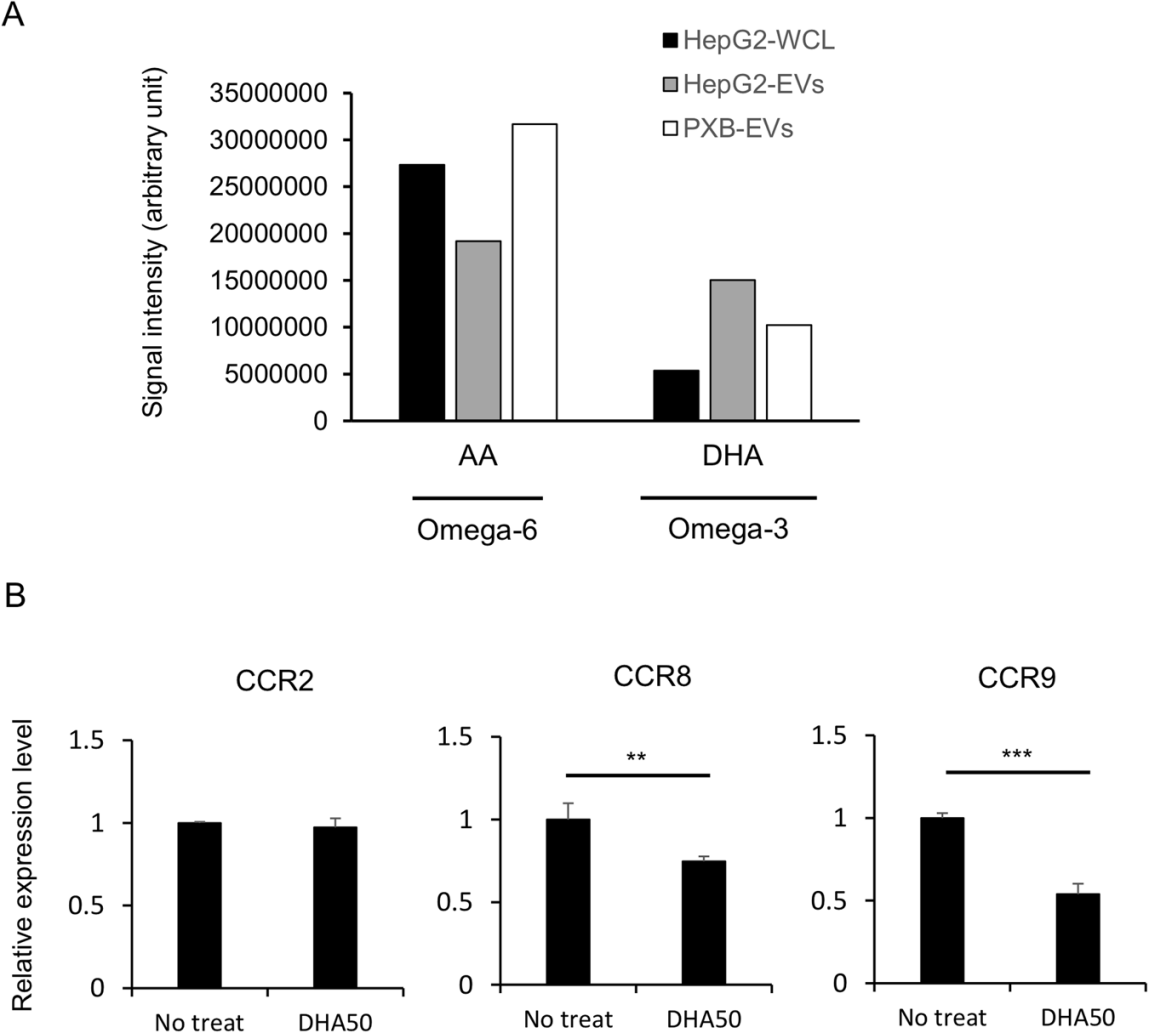
DHA was enriched on the EVs and reduced CCR8 and CCR9 expression in BM cells. **A** Abundance of ω6 and ω3-bound phospholipids (PLs) in whole cells lysate (WLC) or EVs secreted from HepG2 cells and PXB cells. **B** CCR2, CCR8, and CCR9 mRNAs in BM cells 12 h after treatment of 50 μM DHA were measured via RT-qPCR, and each sample was normalized relative to β-actin expression. Data represent the mean ± SD of triplicate experiments. **P < 0.01. ***P < 0.001.

Phopholipid (PL) is one of the most abundant molecules in EVs. Some PLs carry ω6 and ω3 polyunsaturated fatty acids (PUFAs), which are metabolized to bioactive lipid mediators by a series of enzymes including phospholipases. In particular, it was recently reported that PL-bound ω6 and ω3 PUFAs in EVs modulate inflammatory responses on the recipient cells by converting to lipid mediators [22, 23]. To investigate the molecular mechanism underlying the therapeutic effect of HepG2-EVs on ALI, quantitative analysis of PL-bound ω6 (arachidonic acid (AA)) and ω3 PUFAs (docosahexaenoic acid (DHA)) in G2EVs was carried out (Fig. 7A). While AA-bound PLs showed no consistent tendency, HepG2-EVs and PXB-EVs comprised more than 2-fold abundance of DHA-bound PLs compared with the originating HepG2 cells, which implied that EV-carrying DHA mediates the therapeutic effect of HepG2-EVs and PXB-EVs on ALI. To corroborate this, we assessed the effect of DHA on the expression of CCR2, CCR8, and CCR9 mRNAs in mouse BM cells (Fig. 7B). Although DHA negligibly affected CCR2 expression, it significantly reduced the expression levels of CCR8 and CCR9 to a roughly similar extent as those decreased by HepG2-EVs and PXB-EVs (Fig. 6). These results indicate that the DHA molecules carried in human hepatocyte-derived EVs might manifest themselves as reduced CCR8 and CCR9 expression levels in the recipient BM cells.

## Discussion

Currently, EVs are extensively assessed to reveal that they act as critical cell to cell communicators leading to cancer, regenerative disease, and inflammatory diseases [24].

**Fig. 8:**
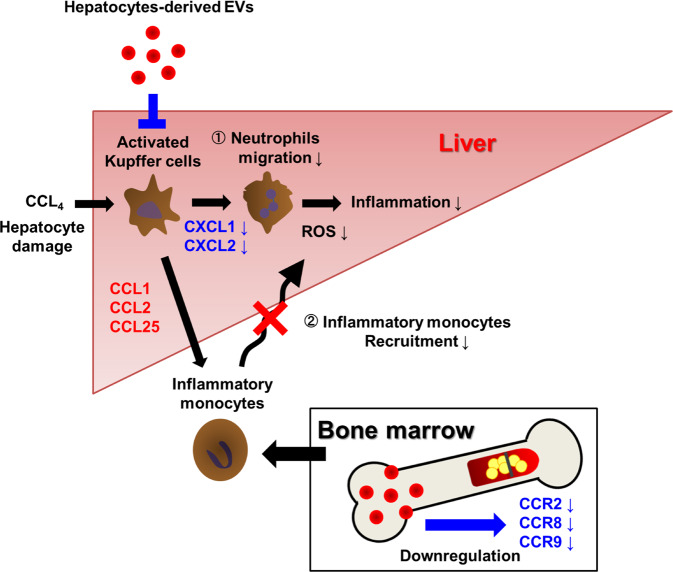
Scheme of Hep-EVs in acute liver injury. Under CCL_4_-induced acute liver injury, KCs are activated and expression of intrahepatic CXCL1, CXCL2 or CCL1, CCL2, and CCL25 is increased, resulting in the recruitment of neutrophils and inflammatory monocytes, further aggravating the liver inflammation. ① Hepatocyte-derived EVs suppress the activation of KCs and decrease CXCL1 and CXCL2. Neutrophil recruitment is inhibited via reduction of intrahepatic CXCL1 and CXCL2. As a result, inflammation is suppressed by reducing ROS production. ② Hep-EV reduces the expression of chemokine receptors CCR2, CCR8, and CCR9 in bone marrow, resulting in suppression of bone marrow-derived monocyte recruitment into the liver.

In the present study, we demonstrated that EVs secreted from human hepatocytes potently attenuated the CCL_4_-induced ALI, which depended on resident KC, by inhibiting the recruitment of neutrophils and monocytes via downregulation of chemokines and chemokine receptor in the BM cells, respectively (Fig. 8). Mechanistically, the characteristic composition of PUFA, particularly DHA, in the EVs is presumably involved in the significant biological process.

Although it has been reported that the EVs secreted from human hepatocytes reveal hepatoprotective effects in vitro [19, 25], their roles in vivo where various types of cells, humoral factors, tissues, and organs locally as well as systemically interact, remains elusive. Accordingly, this is the first study to demonstrate that EVs exert a significant hepatoprotective effect in vivo, which has potent influence both on local and systemic responses.

Most previous studies on hepatocyte EVs have addressed their pathogenic properties when produced by cells that are stressed [26], injured [27, 28], infected [21, 29], or tumorigenic [30] and few studies addressed the biological property of EVs secreted from normal hepatocytes [12, 25]. In the present study, we elucidated the physiological functions of the EVs secreted from hepatocytes under acute injury condition. Hepatocytes and hepatic stellate cells (HSCs) have been studied as target cells of hepatocyte EVs in both acute and chronic injury models, thus revealing that hepatocyte EVs attenuated HSC activation, fibrogenesis, hepatocyte recovery, and expression of inflammatory mediators [12, 25]. In contrast, we identified monocytes/macrophage in the liver as well as BM as a target of EVs in ALI model, which serially revealed that the recruitment of neutrophils releasing ROS, was attenuated via downregulation of CXCL1 and CXCL2 (Figs. 2–4), mainly secreted from macrophages [31]. Though the expression of CCL1 and CCL2 of the KCs in the mice treated with PXB-EVs was significantly less than those with HepG2-EVs and PBS, the monocyte recruitment of those mice with HepG2-EVs and PXB-EVs was equally suppressed (Fig. 5C), suggesting that not only KCs but also other cells composed of the liver might be involved in the monocyte recruitment of CCL_4_-induced ALI. Furthermore, recruitment of monocytes from BM was inhibited due to downregulation of CCR8 and CCR9 by EVs (Fig. 6). Previous studies reported that neither CCR8 nor CCR9 deficient mice developed liver injury despite administration of CCL_4_, thereby indicating that the chemokine receptor expressed on the monocyte is crucial for ALI [5, 32]. Collectively, the downregulation of CCR8 and CCR9, which occurred in the BM by the hepatocyte-EVs, might play a pivotal role in the amelioration of ALI by hepatocyte-EVs.

EVs are now extensively studied due to their potent biological functions. Since the main mechanism of their functions is transfer of their cargo between cells, numerous studies investigated these cargos such as small RNAs, proteins, and DNAs to reveal that they exert functions in the target cells incorporated EVs. Conversely, as few studies investigated about the lipids which have been reported to be enriched in EVs as well, the roles remain elusive. We previously elucidated that the PS exposed on the EVs is indispensable for their incorporation by macrophages [33], suggesting that PL plays crucial roles in EVs. The lipidomics analysis revealed that the composition of PL in the Hep-EVs is characteristic. Remarkably, the PUFA in the sn-2 position of PLs was unique. ω3 PUFAs, DHA was more abundant in HepG2-EVs and PXB-EVs than in HepG2 cells (Fig. 7A). As DHA was converted to immunoregulatory lipid mediators such as resolvin and protectin, it raised the hypothesis that these ω3 PUFA dominant profiles of PL in the EVs might be involved in the hepatoprotective effects. The ω3 PUFA derived lipid mediator downregulates the chemokine receptors on neutrophils in order to inhibit the migration resulting in convergence of inflammation [34], which implied that downregulation of CCR8 and CCR9 is mainly expressed on monocyte by DHA and may be involved in a series of functions for convergence of inflammation, by inhibiting the migration of the BM monocyte to the liver, which is mostly responsible for amelioration of ALI. Actually, DHA has been reported to have therapeutic efficacy for ALI [35]. Though we focus on DHA on sn-2 position of PLs, it is possible that free DHA associated EVs is involved in this mechanism as well.

The reason behind EVs derived from hepatocytes having potent immunotolerative and hepatoprotective functions remains unclear. It is speculated that the liver is a central immunological organ in the human body that is exposed to large amounts of circulating antigens and endotoxins from gut microbiota. To maintain liver homeostasis, the liver employs multiple mechanisms to suppress immune responses and create tolerance [36]. Liver sinusoidal endothelial cells (LSECs), KCs, and dendritic cells play pivotal role in initiating and shaping liver immune responses, through antigen presentation, and cytokine and chemokine excretion along with neutrophils, B and T lymphocytes, and natural killer (NK) cells that circulate in the hepatic sinusoids [37]. Therefore, our results suggested that the liver might employ EVs secreted by hepatocytes to suppress the immune response and create tolerance in order to maintain liver homeostasis. We focused on PL but other factors such as miRNAs, protein, and lipids other than PL might have crucial roles in inducing immunotolerance and hepatoprotection.

MSCs are fibroblast‐like, adherent, immunomodulatory, and multipotent cells. Recent studies indicated that MSCs are a promising therapeutic approach for promoting liver regeneration and repairing liver injury by cell migration into liver sites, hepatogenic differentiation, immunoregulation, and paracrine mechanism [38, 39]. Despite their beneficial properties, there are several limitations to the use of MSCs as cellular therapies. These include the potential for aberrant differentiation, tumor formation [40], and low engraftment [41]. The underlying concerns of the potential for tumor formation or differentiation into undesirable cell types have hindered the adoption and use of MSC-based therapeutic approaches [42]. Therefore, cell-free-based therapy using MSC-EVs has gained immense attention because MSC-EVs contribute to liver injury repair via paracrine mechanism and are safer, cheaper, and more effective [43]; however, they are difficult to culture for longer time periods, and it is costly to keep a fresh supply of EVs. In the present study, we demonstrated that the immunosuppressive effect of HepG2-EVs, human hepatocarcinoma cell line, was comparable to that of EVs secreted from MSCs. HepG2 is easy to culture for longer duration and has the advantage of providing fresh EVs at any time at low cost. Furthermore, continued administration of HepG2-EV to the mice for 3 months did not cause any pathological changes in the mice (Fig. S5). Hence, it could be a potential therapeutic alternative to MSC-EV. For further safety, HepG2-EVs should be administered over a longer duration and the systemic effects should be studied in detail. In this study, the therapeutic effect of the EVs collected from the human cells were tested using the mice CCL_4_-induced liver injury. In order to exclude the affect by species crossover, further study using human liver such as ALI model using chimeric TK-NOG mice will be needed [44].

In summary, hepatocyte EVs have hepatoprotective effects in vivo that are associated with reduced levels of hepatic neutrophils and monocytes, and attenuated expression of inflammatory mediators. These novel findings indicate that hepatocyte EVs are promising candidates as an innovative therapy for liver injury.

## Materials and methods

### Ethics statement

This study was performed in strict accordance with the ethical guidelines of the Declaration of Helsinki and the use of human material was approved by the local ethics committees of Tokai University. Studies on mice were conducted in accordance with both the Guidelines for Animal Experimentation of the Japanese Association for Laboratory Animal Science and the recommendations in the Guide for the Care and Use of Laboratory Animals of the National Institutes of Health.

### Animals

Female C57BL/6 mice 6 weeks old purchased from Nihon Clea. All feeding and experimental procedures were carried out under specific pathogen-free conditions at the animal experiment center of Tokai University. The animals were housed at controlled temperature with 12-h light-dark cycles, fed standard mice chow pellets, had access to water from bottles, and were acclimatized before being studied. All mice were used according to guidelines for experimental animal use specified by the Tokai University.

### Mouse experiments

Six-week-old female mice were injected with hepatocyte-derived EVs (30 μg) or phosphate-buffered saline (PBS) in the tail vein, and after 3 h, 2 ml/kg body weight of CCL_4_ (Fuji Film Wako Pure Chemical Industries, Ltd., Osaka, Japan) was subcutaneously injected. Moreover, 48 h after CCL_4_ administration, mice were sacrificed for tissue and blood collection. Furthermore, we performed the clodronate liposome (CL) injection trial using these mice to investigate the effects of macrophages in CCL_4_-induced liver injury mice. Mice were injected with clodronate- or PBS-liposomes (ClodronateLiposomes.org, Amsterdam, Netherlands) in the tail vein. Next, 24 h after CL injection, the aforementioned procedure was performed.

Thereafter, HepG2-derived EV (30 μg) was injected intraperitoneally for 3 months every week to assess its safety.

Furthermore, the blood samples were centrifuged at 3000 rpm for 15 min at 4 °C to obtain the serum. The livers were rapidly dissected and were snap-frozen and stored at −80 °C before analysis. Eventually, serum levels of glutamic-oxaloacetic transaminase (GOT) and glutamic-pyruvic transaminase (GPT) were measured using an automated chemical analyzer, SPOTCHEM EZ (Arkray, Kyoto, Japan).

### Kupffer cell isolation

The Kupffer cells were isolated by differential centrifugation on an Opti-Prep (Sigma-Aldrich). This protocol was modified from previous reports of isolation Kupffer cells [45, 46]. In particular, the inferior vena cava was cannulated and perfused with 0.5 mM EGTA solution which is added to HBSS without calcium and magnesium, followed by the perfusion buffer (0.4% collagenase type I in HBSS buffer) with 37 °C 5 min. After perfusion, liver was chopped and digested with the digestion buffer (0.4% collagenase type I in MEM Alpha (Life Technologies, Carlsbad, CA with 1% DNase I and 2% FBS) at 37 °C for 20 min with shaking. The cell suspension was filtered through a 100 μm cell strainer and re-suspended to HBSS (Life Technologies) with 2% FBS. Three hundred microliters of cell suspension were collected to isolate bulk liver. Other cell suspensions were centrifuged at 50 × *g* for 1 min at 4 °C to precipitate hepatocytes. The supernatant was centrifuged at 400 × *g* 7 min. 4 °C to obtain hepatic non-parenchymal cells. Pellet was suspended in 15% Opti-Prep and centrifuged at 4 °C for 21 min at 1400 × *g*. The cells included Kupffer cells were collected from the interface. Cells were further purified via subsequent flow cytometric sorting with PI (Bioregends, 421301, San Diego, CA) and CD45-PE (Bioregends, 103105), CD31-FITC (Bioregends, 102405), F4/80-APC (Bioregends, 123115) staining for Kupffer cells using BD FACSMelody cell sorter (Becton Dickinson and Company, New Jersey, USA) [47].

### Primary hepatocytes and cell lines

Primary hepatocytes (PXB cells) were purchased from Phoenix Bio Co., Ltd. (Hiroshima, Japan) and plated on type I collagen-coated 24-well plates. PXB cells were incubated with 500 µL of 2% dimethyl sulfoxide-supplemented hepatocyte clonal growth medium in 5% CO_2_ and 95% air at 37 °C. The culture medium was collected and renewed every 4 days. HepG2 cells were cultured in DMEM high glucose (Life Technologies) supplemented with 10% fetal bovine serum (FBS), 100 units/ml of penicillin, and 100 µg/ml of streptomycin (Life Technologies) in 5% CO_2_ and 95% air at 37 °C.

Ultra-high purity human mesenchymal stem cells (REC), which is a cell line of human mesenchymal stem cells (hMSC), were provided by Dr. Matsuzaki [48]. MSC cells were cultured in DMEM Low glucose (Life Technologies) supplemented with 20% fetal bovine serum (FBS), 100 units/ml of penicillin, and 100 µg/ml of streptomycin (Life Technologies), 0.01 mol/l of Hepes (Life Technologies), 20 ng/ml of bFGF (FUJIFILM Wako Pure Chemical Corporation, Osaka, Japan) in 5% CO_2_ and 95% air at 37 °C.

BM cells were collected from C57BL/6j mice (6 weeks old, female), collected BM cells were cultured in RPMI 1640 medium (Life Technologies) supplemented with 10% (v/v) FBS, 100 units/ml of penicillin, and 100 mg/ml of streptomycin.

### EV isolation

EVs were isolated from cell culture supernatant. To remove bovine EVs from the fetal bovine serum (FBS), FBS was ultracentrifuged at 110,000 × *g* for 18 h at 4 °C using a Type 70.1 Ti (Beckman Coulter, Brea, CA). Thereafter, the supernatant was filtered through a 0.22-μm filter (Merck Millipore, KGaA, Darmstadt, Germany). Next, HepG2 cells were cultured in EV-free, high glucose Dulbecco’s modified Eagle medium (DMEM) (Life Technologies) for 3 days. MSCs were cultured in low glucose DMEM (Life Technologies). Primary hepatocytes (PXB cells) were cultured with 500 µL of 2% dimethyl sulfoxide-supplemented hepatocyte clonal growth medium (Phoenix Bio Co., Ltd, Hiroshima, Japan). Culture supernatants were centrifuged at 1500 × *g* for 15 min at room temperature. To thoroughly remove cellular debris, the supernatants were filtered via a 0.22-μm filter. For EV preparation, the filtered supernatants were ultracentrifuged at 110,000 × *g* for 70 min at 4 °C. The pellets were washed with 8 ml of PBS after ultracentrifugation and resuspended in PBS. HepG2-derived EVs were labeled with PKH26 dye (Sigma-Aldrich). Thereafter, the amount of protein in EVs was measured by using BCA protein assay kit (TAKARA, Shiga, Japan). Electron microscope image of EVs was obtained via transmission EM JEM-1400 (JEOL, Tokyo, Japan). The measurement of size and distribution was based on tunable resistive pulse sensing and carried out using a qNano Gold system (Izon Science Ltd, Christchurch, New Zealand), which combined the tunable nanopores with proprietary data capture and analysis software (Izon Control Suite version 3.3.2.2001; Izon Science).

### Materials

Docosahexaenoic Acid (DHA) was purchased from Cayman Chemical (Ann Arbor, MI). DHA was dissolved in 50 µM with absolute ethanol and added to BM cells. Absolute ethanol was used as a control in all experiments.

### Histology and immunohistochemistry

For histologic analyses, fixed tissues were embedded in paraffin or optimum cutting temperature compound (Sakura Finetechnical, Tokyo, Japan) as previously described [21]. Paraffin-embedded tissues were sectioned and stained with hematoxylin-eosin. For immunostaining, frozen sections were stained using the FITC anti-mouse F4/80 (Biolegend, 157309), FITC anti-mouse Gr-1 (Biolegend, 108405). Nuclei were visualized with DAPI (H-1200; Vector Laboratories, Burlingame, CA). As a secondary antibody, anti-rabbit IgG H&L Alexa Fluor 594 (Abcam, ab150080, Cambridge, UK) and APC streptavidin (Biolegend, 405207) was used. The apoptosis of hepatocytes in liver tissue sections was detected by In Situ Cell Death Detection Kit, Fluorescein (11684795910, Roche Applied Science, Germany). The staining protocol was performed according to the manufacturer’s instructions.

DCFDA/H2DCFDA-Cellular ROS Assay Kit (ab113851, Abcam) was used for ROS detection in liver tissue sections, according to the manufacturer’s instructions. DCFDA positive cells were detected with filter set appropriate for fluorescein (FITC).

TUNEL, Gr-1, F4/80, or DAPI positive cells were counted in randomized 10 fields (magnification: ×100 or 200) from 3 mice liver tissues. Ratios of positive cells versus total cell counts were analyzed. Fluorescent intensity of DCFDA stained sections was measured in randomized 10 fields (magnification: ×100) from 3 mice liver tissues. Stained sections were mounted with gold antifade reagent (Life Technologies) and examined using the Zeiss LSM700 scanning laser confocal microscope (Carl Zeiss, Oberkochen, Germany). For positive cells, a representative area had been stored and quantified with Fiji software (derived from NIH ImageJ).

### qRT-PCR

In vivo experiments, liver tissue samples, isolated Kupffer cells, and BM cells were collected and frozen immediately with Sepasol^®^-RNA I Super G (Nacalai Tesque, Kyoto, Japan) and stored at −80 °C until RNA extraction. In vitro experiments, BM cells, were seeded in 6-well plates at a density of 1 × 10^6^ cells per well, and cultured with hepatocytes-derived EVs or DHA in the above culture medium for 12 h. Total RNA isolation was performed according to the protocol provided with the above reagents. Reverse-transcription to cDNA was performed using High-Capacity cDNA Reverse Transcription Kit (Applied Biosystems™, Foster City, CA) according to the manufacturer’s instructions. In vivo experiments, pro-inflammatory cytokine (IL-6, TNF-α, IL-1β), C-X-C motif chemokine ligand (CXCL1, CXCL2), and C-C motif chemokine ligand (CCL1, CCL2, CCL25) were quantified using real-time PCR. In vitro experiments, C-C chemokine receptors (CCR2, CCR8, CCR9) were quantified. In addition, the CLEC4F, the maker of Kupffer cells were applied to confirm isolation Kupffer cells as previously described [45, 47]. PCR was performed using the THUNDERBIRD^®^ SYBR^®^ qPCR Mix (TOYOBO CO., Ltd, Osaka, Japan) and the primer pairs with the following program: initial denaturation at 95 °C for 1 min followed by 40 cycles of amplification at 95 °C for 15 s, annealing at 60 °C for 15 s, and extension at 72 °C for 30 s. The primer pairs were shown in Supporting Table S1.

### Western blot analysis

EV pellets were lysed in RIPA assay buffer (FUJIFILM Wako Pure Chemical Corporation) for 30 min on ice. Protein samples were electrophoresed on a 10% SDS–polyacrylamide gel and blotted onto polyvinylidene difluoride membranes (Bio-Rad Laboratories, Hercules, CA). The membranes were washed with TBS-Tween and were shaken in Can Get Signal PVDF Blocking Reagent (TOYOBO) at room temperature for 1 h. After washed, the membranes were incubated with primary antibodies diluted in Can Get Signal Solution 1 (TOYOBO) at room temperature for 1 h. After washed, the membrane was incubated with secondary antibodies conjugated to horseradish peroxidase (HRP) diluted in Can Get Signal Solution 2 (TOYOBO) at room temperature for 1 h. After washed, the membranes were incubated with Immobilon Western Chemiluminescent HRP Substrate (Merck Millipore, KGaA, Darmstadt, Germany) for a few seconds, and chemiluminescence was detected using the ChemiDoc Touch system (Bio-Rad). The following antibodies were used: mouse anti-CD63 (Santa Cruz Biotechnology, Santa Cruz, CA) and sheep anti-mouse IgG HRP (Sigma).

### Phospholipid profiling

Lipids were extracted from the EVs by adding 10-fold methanol containing 0.1% (v/v) formic acid, and were then centrifuged at 12,000 × *g* for 5 min. Resultant supernatant was used for phospholipid profiling. The main phospholipids were analyzed using a Shimadzu UHPLC-Nexera system coupled to a LCMS-8050 triple mass spectrometer (Shimadzu, Kyoto, Japan), using Labsolutions (version 5.91) software for data acquisition and analysis. Kinetex C8 column (Phenomenex, Torrance, CA, USA, 2.1 × 150 mm, 2.6 μm) was used for LC separation. Column temperature was controlled at 45 °C. The mobile phases A and B comprised 20 mM ammonium formate in water and 50% (v/v) 2-propanol in acetonitrile, respectively. The gradient was as follows: 20% B at 0 min, 20–40% B at 1–2 min, 40–92.5% B at 2–25 min, 92.5–100% B at 25–26 min, 100% B at 26–35 min, 100–20% B at 35–35.1 min, and 20% B at 38 min. The flow rate of mobile phase was 0.3 mL/min and the injection volume was 50 μL. An electrospray source was employed in positive (for PE, PC analysis) and negative ion mode (for PI and PS analysis). Moreover, ultra-pure argon (99.9999%) was used as the collision gas. Needle voltage was set at 4.0 kV. Phospholipid analysis was carried out according to LC/MS/MS MRM library for Phospholipid (PL) profiling (Shimadzu). Furthermore, the initial step was carried out to classify PLs [phosphatidylcholine (PC), phosphatidylethanolamine (PE), phosphatidylglycerol (PG), phosphatidylinositol (PI), phosphatidylserine (PS), lysoPC, lysoPE, lysoPG, lysoPI, lysoPS] except for phosphatidic acids by detecting characteristic fragment ions (PC and lysoPC, *m*/*z* 184 in positive ion mode; PE and lysoPE, *m*/*z* M-141.0 in positive ion mode; PG and lysoPG, *m*/*z* M-172.1 in positive ion mode; PI and lysoPI, *m*/*z* 241 in negative ion mode; PS and lysoPS, *m*/*z* 184.10 in positive ion mode). Subsequently, the fatty acyl moiety of the PLs was identified by MRM in the negative ion mode to quantify individual PL molecules. The equal amount of standard compounds of each PL class, namely PC(17:0/20:4), PE(17:0/20:4), PG(17:0/20:4), PI(17:0/20:4), PS(17:0/20:4), lysoPC(17:0), lysoPE(17:1), lysoPG(17:1), lysoPI(17:1), and lysoPS(17:1), were analyzed to normalize the signal intensities among different PL classes.

### Statistical analysis

All statistical analyses were performed using R software. Differences between means were evaluated using one-way ANOVA and subsequent Tukey’s honest significant difference (HSD) method for multiple comparisons or student’s *t*-test for the analysis of the significance between two groups. Values of *p* < 0.05 were considered as statistically significant. Data are presented as the mean ± SD.

## Supplementary information

Supplemental Material

## Data Availability

Data sharing is not applicable to this article as no datasets were generated or analyzed during the current study.

## References

[1] Kubes P, Jenne C (2018). Immune responses in the liver. Annu Rev Immunol.

[2] Stravitz RT, Lee WM (2019). Acute liver failure. Lancet.

[3] Ryu KH, Kim SY, Kim YR, Woo SY, Sung SH, Kim HS (2014). Tonsil-derived mesenchymal stem cells alleviate concanavalin A-induced acute liver injury. Exp Cell Res.

[4] Amiya T, Nakamoto N, Chu PS, Teratani T, Nakajima H, Fukuchi Y (2016). Bone marrow-derived macrophages distinct from tissue-resident macrophages play a pivotal role in Concanavalin A-induced murine liver injury via CCR9 axis. Sci Rep.

[5] Nakamoto N (2012). CCR9+ macrophages are required for acute liver inflammation in mouse models of hepatitis. Gastroenterology.

[6] Zigmond E, Samia-Grinberg S, Pasmanik-Chor M, Brazowski E, Shibolet O, Halpern Z (2014). Infiltrating monocyte-derived macrophages and resident kupffer cells display different ontogeny and functions in acute liver injury. J Immunol.

[7] Rolando N, Wade J, Davalos M, Wendon J, Philpott-Howard J, Williams R (2000). The systemic inflammatory response syndrome in acute liver failure. Hepatology.

[8] Wang J, Ren H, Yuan X, Ma H, Shi X, Ding Y (2018). Interleukin-10 secreted by mesenchymal stem cells attenuates acute liver failure through inhibiting pyroptosis. Hepatol Res.

[9] Chen L, Xiang B, Wang X, Xiang C (2017). Exosomes derived from human menstrual blood-derived stem cells alleviate fulminant hepatic failure. Stem Cell Res Ther.

[10] Lou G, Yang Y, Liu F, Ye B, Chen Z, Zheng M (2017). MiR-122 modification enhances the therapeutic efficacy of adipose tissue-derived mesenchymal stem cells against liver fibrosis. J Cell Mol Med.

[11] Jiang W, Tan Y, Cai M, Zhao T, Mao F, Zhang X (2018). Human umbilical cord MSC-derived exosomes suppress the development of CCl. Stem Cells Int.

[12] Li X, Chen R, Kemper S, Brigstock DR (2019). Extracellular vesicles from hepatocytes are therapeutic for toxin-mediated fibrosis and gene expression in the liver. Front Cell Dev Biol.

[13] Raposo G, Stoorvogel W (2013). Extracellular vesicles: exosomes, microvesicles, and friends. J Cell Biol.

[14] Kowal J, Tkach M, Théry C (2014). Biogenesis and secretion of exosomes. Curr Opin Cell Biol.

[15] Jia S, Zocco D, Samuels ML, Chou MF, Chammas R, Skog J (2014). Emerging technologies in extracellular vesicle-based molecular diagnostics. Expert Rev Mol Diagn.

[16] Imani Fooladi AA, Mahmoodzadeh, Hosseini H (2014). Biological functions of exosomes in the liver in health and disease. Hepat Mon.

[17] Burrello J, Monticone S, Gai C, Gomez Y, Kholia S, Camussi G (2016). Stem cell-derived extracellular vesicles and immune-modulation. Front Cell Dev Biol.

[18] Li X, Li C, Zhang L, Wu M, Cao K, Jiang F (2020). The significance of exosomes in the development and treatment of hepatocellular carcinoma. Mol Cancer.

[19] Holman NS, Church RJ, Nautiyal M, Rose KA, Thacker SE, Otieno MA (2019). Hepatocyte-derived exosomes promote liver immune tolerance: possible implications for idiosyncratic drug-induced liver injury. Toxicol Sci.

[20] Tacke F, Zimmermann HW (2014). Macrophage heterogeneity in liver injury and fibrosis. J Hepatol.

[21] Kakizaki M, Yamamoto Y, Otsuka M, Kitamura K, Ito M, Kawai HD (2020). Extracellular vesicles secreted by HBV-infected cells modulate HBV persistence in hydrodynamic HBV transfection mouse model. J Biol Chem.

[22] Sagini K, Costanzi E, Emiliani C, Buratta S, Urbanelli L (2018). Extracellular vesicles as conveyors of membrane-derived bioactive lipids in immune system. Int J Mol Sci.

[23] Cianciaruso C, Beltraminelli T, Duval F, Nassiri S, Hamelin R, Mozes A (2019). Molecular profiling and functional analysis of macrophage-derived tumor extracellular vesicles. Cell Rep.

[24] van Niel G, D’Angelo G, Raposo G (2018). Shedding light on the cell biology of extracellular vesicles. Nat Rev Mol Cell Biol.

[25] Nojima H, Freeman CM, Schuster RM, Japtok L, Kleuser B, Edwards MJ (2016). Hepatocyte exosomes mediate liver repair and regeneration via sphingosine-1-phosphate. J Hepatol.

[26] Verma VK, Li H, Wang R, Hirsova P, Mushref M, Liu Y (2016). Alcohol stimulates macrophage activation through caspase-dependent hepatocyte derived release of CD40L containing extracellular vesicles. J Hepatol.

[27] Seo W, Eun HS, Kim SY, Yi HS, Lee YS, Park SH (2016). Exosome-mediated activation of toll-like receptor 3 in stellate cells stimulates interleukin-17 production by γδ T cells in liver fibrosis. Hepatology.

[28] Kakazu E, Mauer AS, Yin M, Malhi H (2016). Hepatocytes release ceramide-enriched pro-inflammatory extracellular vesicles in an IRE1α-dependent manner. J Lipid Res.

[29] Cobb DA, Kim OK, Golden-Mason L, Rosen HR, Hahn YS (2018). Hepatocyte-derived exosomes promote T follicular regulatory cell expansion during hepatitis C virus infection. Hepatology.

[30] Pascut D, Pratama MY, Vo NVT, Masadah R, Tiribelli C (2020). The crosstalk between tumor cells and the microenvironment in hepatocellular carcinoma: the role of exosomal microRNAs and their clinical implications. Cancers.

[31] Marra F, Tacke F (2014). Roles for chemokines in liver disease. Gastroenterology.

[32] Heymann F, Hammerich L, Storch D, Bartneck M, Huss S, Rüsseler V (2012). Hepatic macrophage migration and differentiation critical for liver fibrosis is mediated by the chemokine receptor C-C motif chemokine receptor 8 in mice. Hepatology.

[33] Higuchi H, Yamakawa N, Imadome KI, Yahata T, Kotaki R, Ogata J (2018). Role of exosomes as a proinflammatory mediator in the development of EBV-associated lymphoma. Blood.

[34] Murakami M (2011). Lipid mediators in life science. Exp Anim.

[35] Enguita M, Razquin N, Pamplona R, Quiroga J, Prieto J, Fortes P (2019). The cirrhotic liver is depleted of docosahexaenoic acid (DHA), a key modulator of NF-κB and TGFβ pathways in hepatic stellate cells. Cell Death Dis.

[36] Jenne CN, Kubes P (2013). Immune surveillance by the liver. Nat Immunol.

[37] Heymann F, Tacke F (2016). Immunology in the liver-from homeostasis to disease. Nat Rev Gastroenterol Hepatol.

[38] Kang SH, Kim MY, Eom YW, Baik SK (2020). Mesenchymal stem cells for the treatment of liver disease: present and perspectives. Gut Liver.

[39] Hu C, Wu Z, Li L (2020). Pre-treatments enhance the therapeutic effects of mesenchymal stem cells in liver diseases. J Cell Mol Med.

[40] Volarevic V, Markovic BS, Gazdic M, Volarevic A, Jovicic N, Arsenijevic N (2018). Ethical and safety issues of stem cell-based therapy. Int J Med Sci.

[41] Kurtz A (2008). Mesenchymal stem cell delivery routes and fate. Int J Stem Cells.

[42] Herberts CA, Kwa MS, Hermsen HP (2011). Risk factors in the development of stem cell therapy. J Transl Med.

[43] Hu C, Zhao L, Zhang L, Bao Q, Li L (2020). Mesenchymal stem cell-based cell-free strategies: safe and effective treatments for liver injury. Stem Cell Res Ther.

[44] Suemizu H, Hasegawa M, Kawai K, Taniguchi K, Monnai M, Wakui M (2008). Establishment of a humanized model of liver using NOD/Shi-scid IL2Rgnull mice. Biochem Biophys Res Commun.

[45] Bonnardel J, T’Jonck W, Gaublomme D, Browaeys R, Scott CL, Martens L (2019). Stellate cells, hepatocytes, and endothelial cells imprint the Kupffer cell identity on monocytes colonizing the liver macrophage niche. Immunity.

[46] Choi WM, Kim HH, Kim MH, Cinar R, Yi HS, Eun HS (2019). Glutamate signaling in hepatic stellate cells drives alcoholic steatosis. Cell Metab.

[47] Lynch RW, Hawley CA, Pellicoro A, Bain CC, Iredale JP, Jenkins SJ (2018). An efficient method to isolate Kupffer cells eliminating endothelial cell contamination and selective bias. J Leukoc Biol.

[48] Mabuchi Y, Morikawa S, Harada S, Niibe K, Suzuki S, Renault-Mihara F (2013). LNGFR(+)THY-1(+)VCAM-1(hi+) cells reveal functionally distinct subpopulations in mesenchymal stem cells. Stem Cell Rep.

