# Supplementary material for: Human hepatocyte-derived extracellular vesicles attenuate the carbon tetrachloride-induced acute liver injury in mice
Source: Cell Death Dis. 2021 Oct 27;12(11):1010. doi: 10.1038/s41419-021-04204-7 (PMC8551237; doi:10.1038/s41419-021-04204-7)
Supplement: Supplementary file 1 — Supplemental Material [file 41419_2021_4204_MOESM1_ESM.docx]

**Supporting Information**

**Human hepatocytes-derived extracellular vesicles attenuate the carbon tetrachloride-induced acute liver injury in mice**

Masatoshi Kakizaki^1,2)^, Yuichiro Yamamoto^1,2)^, Shunya Nakayama^1,2)^, Kazuaki Kameda^1,2,3)^, Etsuko Nagashima^1,2)^, Masatoshi Ito^4)^, Takashi Suyama^5)^, Yumi Matsuzaki^5)^, Tetsuhiro Chiba^6)^, Hideaki Sumiyoshi^1,7,8)^, Yutaka Inagaki^1,7,8)^ and Ai Kotani^1,2)^

^1^Department of Innovative Medical Science, Tokai University School of Medicine,

Kanagawa, Japan

^2^Division of Hematological Malignancy, Institute of Medical Sciences, Tokai University, Kanagawa, Japan

^3^Division of Hematology, Jichi Medical University Saitama Medical Center, Saitama, 330-8503, Japan

^4^Support Center for Medical Research and Education, Tokai University School of Medicine, Kanagawa, Japan

^5^Department of Life Science, Shimane University Faculty of Medicine, Izumo, Shimane, Japan

^6^Department of Gastroenterology, Chiba University, Graduate School of Medicine,

Inohana, Chuo-ku, Chiba, Japan

^7^Center for Matrix Biology and Medicine, Graduate School of Medicine, Tokai University, Kanagawa, Japan

^8^Institute of Medical Sciences, Tokai University, Kanagawa, Japan

***To whom correspondence should be addressed**

Ai Kotani, M.D., Ph.D.

Department of Innovative Medical Science, Tokai University School of Medicine,

Kanagawa, Japan

Division of Hematological Malignancy, Institute of Medical Sciences, Tokai University, Kanagawa, Japan

Tel.: 81.463-93-1121

E-mail: aikotani@k-lab.jp

**Supporting Figures**

Supporting Figure. 1 Characterization of EVs secreted from HepG2, PXB and REC cells.

**
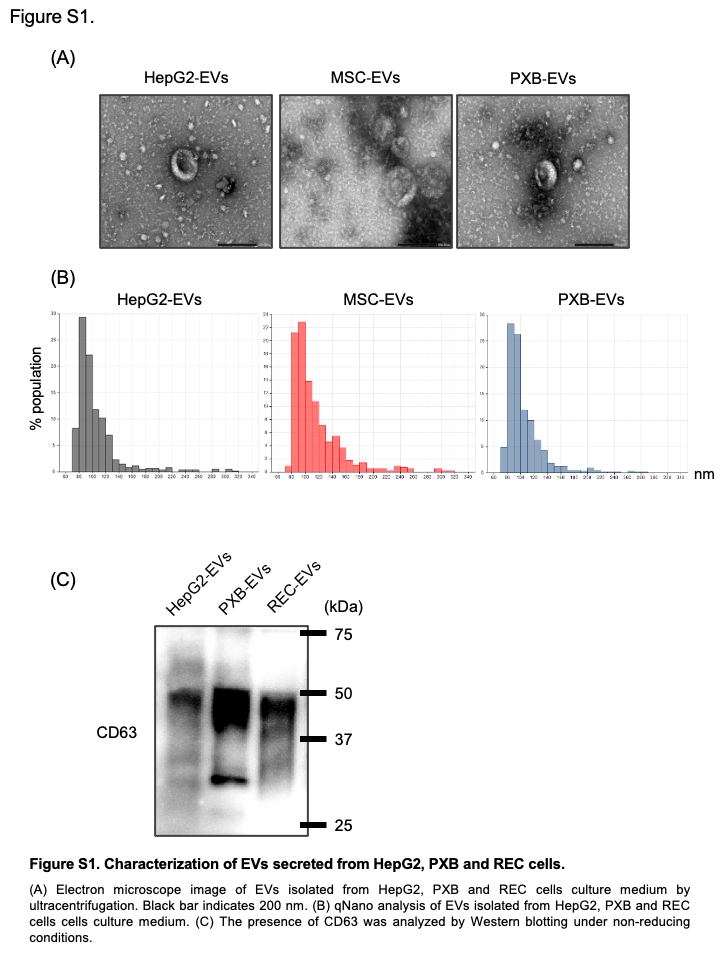
**

(A) Electron microscope image of EVs isolated from HepG2, PXB and REC cells culture medium by ultracentrifugation. Black bar indicates 200 nm. (B) qNano analysis of EVs isolated from HepG2, PXB and REC cells cells culture medium. (C) The presence of CD63 was analyzed by Western blotting under non-reducing conditions.

Supporting Figure. 2 Hepatoprotective effects after Hep-EVs treatment.


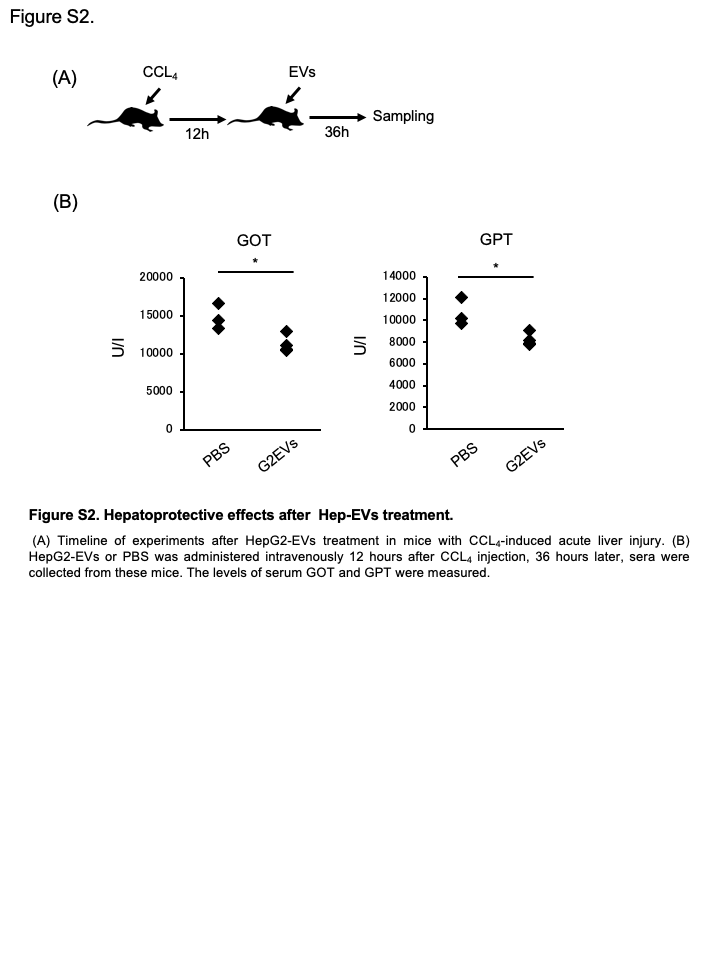


(A) Timeline of experiments after HepG2-EVs treatment in mice with CCL_4_-induced acute liver injury. (B) HepG2-EVs or PBS was administered intravenously 12 hours after CCL_4_ injection, 36 hours later, sera were collected from these mice. The levels of serum GOT and GPT were measured.

Supporting Figure. 3 Kupffer cell isolation from bulk liver


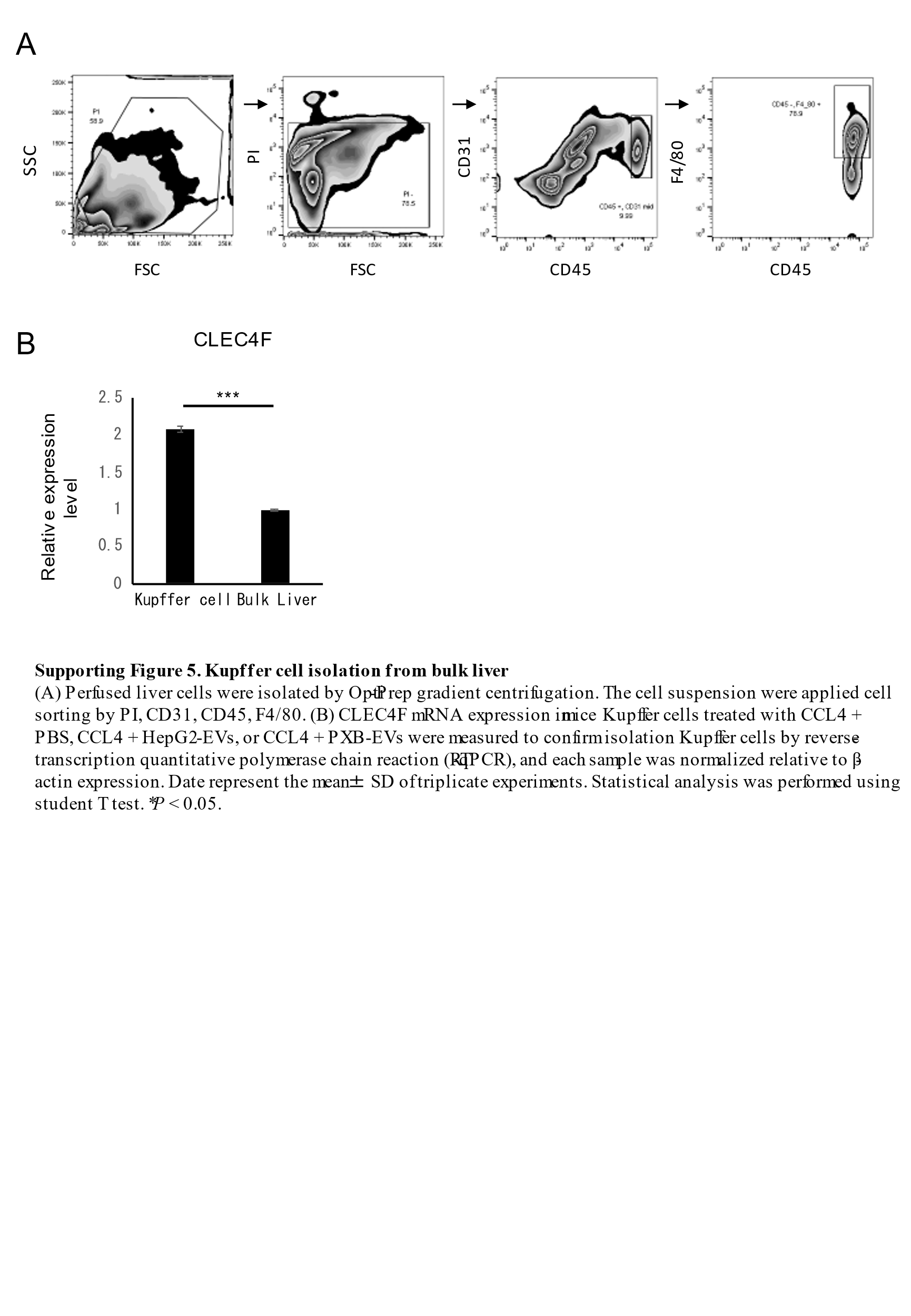


(A) Perfused liver cells were isolated by Opti-Prep gradient centrifugation. The cell suspension were applied cell sorting by PI, CD31, CD45, F4/80. (B) CLEC4F mRNA expression in mice Kupffer cells treated with CCL4 + PBS, CCL4 + HepG2-EVs, or CCL4 + PXB-EVs were measured to confirm isolation Kupffer cells by reverse-transcription quantitative polymerase chain reaction (RT-qPCR), and each sample was normalized relative to β-actin expression. Date represent the mean ± SD of triplicate experiments. Statistical analysis was performed using student T test. *P < 0.05.

Supporting Figure. 4 Effects of Hep-EVs on the expression of matrix metalloproteinases (MMP-9, MMP-12) in CCL_4_-induced acute liver injury


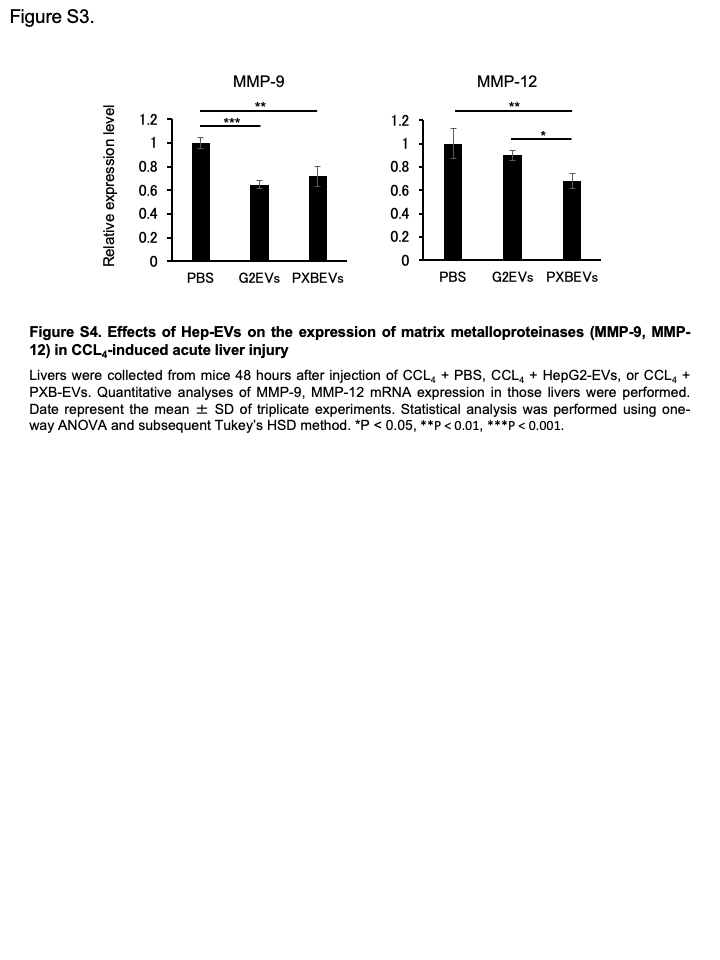


Livers were collected from mice 48 hours after injection of CCL_4_ + PBS, CCL_4_ + HepG2-EVs, or CCL_4_ + PXB-EVs. Quantitative analyses of MMP-9, MMP-12 mRNA expression in those livers were performed. Date represent the mean ± SD of triplicate experiments. Statistical analysis was performed using one-way ANOVA and subsequent Tukey’s HSD method. *P < 0.05, **P < 0.01, ***P < 0.001.

Supporting Figure. 5 Effects on the liver of mice treated with HepG2-EVs for 3 months

**
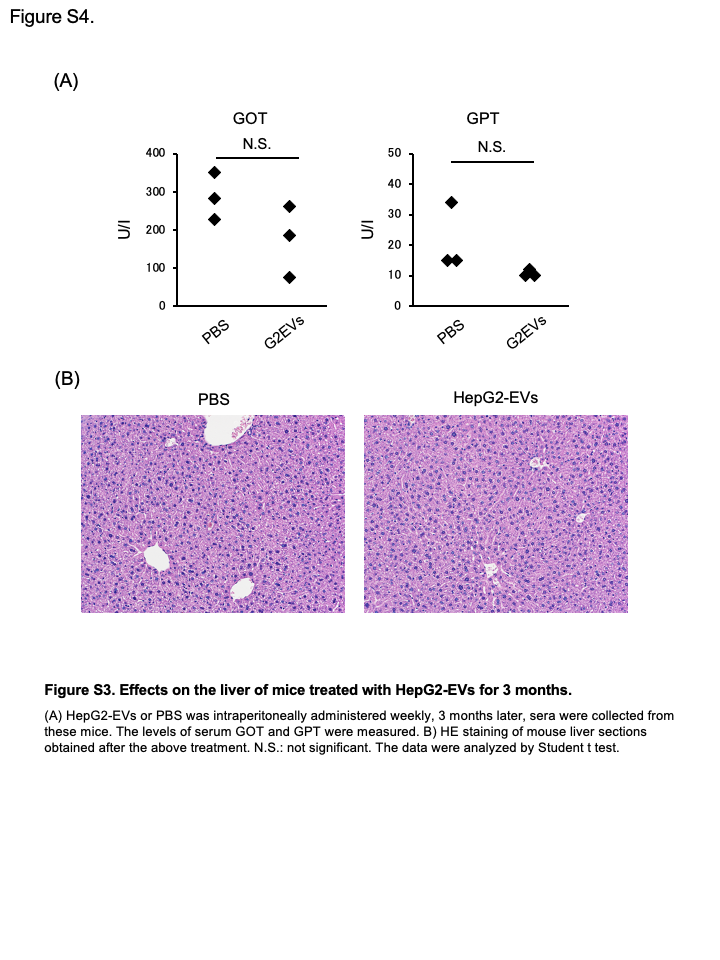
**

(A) HepG2-EVs or PBS was intraperitoneally administered weekly, 3 months later, sera were collected from these mice. The levels of serum GOT and GPT were measured. B) HE staining of mouse liver sections obtained after the above treatment. N.S.: not significant. The data were analyzed by Student t test.

**Supporting Tables**

Supporting Table S1. Primer sequences used for quantification.

| Gene symbol | Forward primer | Reverse primer |
| --- | --- | --- |
| *β-actin* | 5′-CATGAAGTGTGACGTTGACATC-3′ | 5′-ATGATCTTGATCTTCATGGTGCTA-3′ |
| *IL-6* | 5′-TGGAGTCACAGAAGGAGTGGCTAAG-3′ | 5′-CTGACCACAGTGAGGAATGTCCA-3′ |
| *TNF-α* | 5′-CCACCACGCTCTTCTGTCTA-3′ | 5′-TGGGCTACAGGCTTGTCA-3′ |
| *IL-1β* | 5′-GGATGAGGACATGAGCACCT-3′ | 5′-AGCTCATATGGGTCCGACAG-3′ |
| *CXCL1* | 5′-CAGAGCCTCTAACCAGTTCCA-3′ | 5′-TGGGATCATGGTGCTGTG-3′ |
| *CXCL2* | 5′-AAAGGCAAGGCTAACTGACCT-3′ | 5′-CTTTGGTTCTTCCGTTGAGG-3′ |
| *CCL1* | 5′-TCACCATGAAACCCACTGC-3′ | 5′-GCTGTCAACATCCTGTATCCAC-3′ |
| *CCL2* | 5′-GGCTGGAGAGCTACAAGAGG-3′ | 5′-TCTTGAGCTTGGTGACAAAAAC-3′ |
| *CCL25* | 5′-GGTTCCATCTGCAGTGGTTT-3′ | 5′-TTTAAGGGGGCCACCAAT-3′ |
| *MMP-9* | 5′-TTCACCGGCTAAACCACCT-3′ | 5′-CATGCATGTGAACATAACCTCA-3′ |
| *MMP-12* | 5′-TTGTGGATAAACACTACTGGAGGT-3′ | 5′-AAATCAGCTTGGGGTAAGCA-3′ |
| *CCR2* | 5′-CCACACCCTGTTTCGCTGTA-3′ | 5′-TGCATGGCCTGGTCTAAGTG-3′ |
| *CCR8* | 5′-TGCGATGTGTAAGGTGGTCTC-3′ | 5′-TGATGGCATAGACAGCGTGG-3′ |
| *CCR9* | 5′-TGTAAGAAAAATAATGTCAGGCAGT-3′ | 5′-CAGAAGGGAAGAGTGGCAAG-3′ |
| *CLEC4F* | 5'-CAACAACTCTGGACACGACAA-3' | 5'-AGCCTCTGGATAGCCACTTG-3' |
